# The Role of Individual Knowledge in Functional Olive Oil Preferences: Does Self-Coherence Lead to Different Health Attributes Perception?

**DOI:** 10.3390/foods9101428

**Published:** 2020-10-09

**Authors:** Giuseppe Di Vita, Alfio Strano, Giulia Maesano, Giovanni La Via, Mario D’Amico

**Affiliations:** 1 Department of Agricultural, Forest and Food Science (Disafa), University of Turin, Largo Braccini, 2, 10095 Grugliasco, Torino, Italy; giuseppe.divita@unito.it; 2 Department of Agriculture, Mediterranean University of Reggio Calabria, Località Feo di Vito, 89122 Reggio Calabria, Italy; astrano@unirc.it; 3 Department of Agriculture, Food and Environment (Di3A), University of Catania, Via S. Sofia 98-100, 95123 Catania, Italy; giovanni.lavia@unict.it (G.L.V.); mario.damico@unict.it (M.D.)

**Keywords:** antioxidant, polyphenols, functional olive oil, healthy food products, subjective knowledge, self-consistency, health claims

## Abstract

This study examined whether health cues influence the choices of olive oil consumers with different degrees of knowledge about the nutritional properties of olive oil. To this end, a direct survey on the consumption of healthy extra-virgin olive oil was implemented by examining the stated preferences of a sample of consumers. Two econometric analyses were carried out to identify the drivers of the consumption of olive oil with high polyphenol content. The logistic model was chosen as the most suitable method to answer the research questions. The results revealed a general consensus among consumers regarding the beneficial properties of olive oil consumption. Moreover, the findings show that different degrees of individual knowledge act as distinctive drivers in influencing the health perception of olive oil consumers. Finally, this study verified that, even for healthy foods, consumers’ choices are strongly dependent on their own self-coherence. As a consequence, consumers’ knowledge or beliefs that orientate their attitudes are influenced by different motivations and attributes.

## 1. Introduction

Despite the wide consensus among nutritionists, biologists, doctors, and nutraceutical experts on the meaning of the term ‘healthy’, consumers do not always associate this term with food or fully understand its meaning in reference to food [1]. In many cases, consumers perceive the healthiness of food solely in relation to its nutritional content [1].

Healthiness perceptions can be attributed to single products or to components of one’s diet. The perceived healthiness of food tends to decrease in the presence of high sodium content or additives, fats, and carbohydrates [2]. On the other hand, food that has high protein content is often deemed healthy [1,2,3].

Healthiness is now a highly popular topic among consumers and the use of the term is regulated by the European Commission, which associates it with ‘functional’ food. However, consumers perceive the term ‘healthy’ in a subjective manner. The definition of the term ‘healthy’ is not always consistent, and nuances and subjective conceptualizations persist among consumers [1]. Scholars and researchers have paid significant attention to health claims, and particular emphasis has been placed on labels reporting the nutrition and healthiness of foods [4,5,6], that aim to allow consumers to also better identify functional foods, even in the absence of direct or specific knowledge. Recent studies on consumers’ perceptions of the healthiness of olive oil have indicated that consumers feel that it is a naturally healthy food. However, although olive oil consumers are aware of the health properties of olive oil, they are not familiar with the health benefits and roles of each specific nutritional and intrinsic compound of the product [7,8].

Research on olive oil consumers has shown that consumers’ personal traits have a strong effect on their preferences [9] and studies have shown that the consumer’s choice of a healthier olive oil depends both on their subjective and nutritional knowledge [7,10]. Indeed, subjective knowledge influences individuals’ choices according to their level of information. In parallel with label detection, the subjective or perceived knowledge differs as environmental stimuli are consistent with consumers’ subjective information and educational level and finally with their self-consistency. Consequently, differences in consumers’ individual knowledge and access to information give them different perceptions of the healthiness of olive oil.

Consumers have different levels of knowledge based on their beliefs (related to their education, occupation, and level of health awareness), the available information (from sources such as magazines, television, social networks, etc.), and their habits and curiosity (personal interest, attendance at sensory analysis courses, etc.). Subjective knowledge also strongly influences consumers’ store selectivity and product characterisation. For example, subjective knowledge may make consumers place food into more or less healthy categories according to their quality choices [11]. High-knowledge consumers are also typically more certain regarding the quality of their personal choices [12].

Prior research has identified the effects of individual knowledge on ‘nutrition search selectivity and choice’, showing that consumers’ self-consistency helps, directs and orientates them [11,13]. However, the role of health cues in consumers’ olive oil preferences is still under researched. Even though consumers’ attention to the healthiness of olive oil is growing progressively and previous studies have pointed to the importance of functional olive oil in consumers’ preferences [14], there is a lack of clarity regarding the role of information in healthy food choices and whether and how the consumption of functional products is influenced by individual knowledge.

To the authors’ knowledge, no study has investigated whether self-consistency could lead to a different perception towards the health attributes of the same product, and whether preference varies as the subjective knowledge of the consumer varies. As a consequence, this paper intends to fulfil the gap in the existing literature.

The hypothesis of the present study is that individual knowledge has different effects on consumers’ preferences for healthy olive oil. The study aimed to explore whether consumers’ choices for healthy olive oil were dependent on their subjective knowledge, beliefs and own self-coherence that orientate their attitudes, and whether these attitudes are influenced by personal motivations and sensory attributes.

Given that the experts’ attention is progressively focusing on the role that polyphenol content has in olive oil consumer’s healthiness, this paper thus presents a case study of consumers’ attitudes towards a functional olive oil with a high polyphenol content. Regulation (EC) No 1924/2006 and subsequent amendments identifies ‘functional health olive oils’ according to their polyphenol, oleic acid, and vitamin E content. Regarding polyphenol content, an olive oil can be defined as functional if it ‘contains at least 5 mg of hydroxytyrosol and its derivatives (e.g., oleuropein complex and tyrosol) per 20 g of olive oil’.

In particular, this study tested the hypothesis that preferences for healthier olive oils with a higher polyphenol content change according to the consumers’ levels of individual knowledge and self-consistency. In this study, the variables that influence the consumers’ interest in and knowledge about the nutritional and health properties of extra-virgin olive oil were identified and analysed using a logit model and evaluating the marginal effects. This study examined whether health cues influence the choices of olive oil consumers with different degrees of knowledge about the nutritional properties of olive oil. To this end, this study tries to answer the following research question: which variables influence low and high-knowledge consumers’ attitudes towards functional olive oils?

## 2. Literature on Antioxidants and Health Claims on Olive Oil Bottles

This literature review aimed to identify the main contributions of the literature on olive oil consumer’s behaviour by analysing the following items: (a) consumers’ perceptions of the healthiness of olive oil and the role played by polyphenol content in olive oil consumers’ preferences; (b) the function of healthiness labels (claims) as drivers of health information for olive oil consumers; and (c) the role of consumers’ knowledge in their food and olive oil choices.

### 2.1. Healthiness of Olive Oil

The existing literature has emphasized the health benefits of the regular consumption of olive oil. The healthiness of the Mediterranean diet is well established, as are the benefits of olive oil for consumers’ health [15]. The benefits of olive oil are mainly attributable to its high oleic acid content as well as its phenolic compounds; both contribute to olive oil’s antioxidant, anti-inflammatory, and antimicrobial properties [15,16].

Consumers’ positive perceptions of the healthiness of olive oil have been confirmed by recent studies on the biological properties of olive oil’s polyphenols, which play a role in the prevention of cardiovascular diseases and cancer and have anti-inflammatory, antimicrobial, and antiviral properties [16,17]. In addition, it has been found that a diet based on high-phenolic olive oil ‘improves ischemic reactive hyperaemia during the postprandial state’ [18].

Within this context, the role of polyphenol content in consumers’ food choices has been of wide interest for scholars and researchers [14,19,20], but consumers’ perceptions of the beneficial properties of antioxidant compounds in olive oil and its high therapeutic potential for the treatment of cardiovascular diseases has scarcely been examined.

Consumers’ perceptions of the healthiness of olive oil was first addressed by Krystallis and Ness, (2005) [21]. The authors identified different olive oil consumer segments, highlighting the group of ‘health-and quality-conscious consumers’. This segment of consumers was also identified in another study that pointed to the importance of nutraceutical information reported on labels and the high interest of extra-virgin olive oil consumers in health claims, finding that single males and elderly individuals seem to be the most sensitive to the product’s healthy characteristics [22]. In this case, a direct association was observed between the nutraceutical properties and the production area [22]. Consumers’ interest in the presence of nutraceutical information on labels was also assessed in another study, which showed that the health claims related to the polyphenol content of olive oil can help signal the ‘healthiest properties’ of extra-virgin olive oils [14]. Nevertheless, in traditional olive oil production countries such as Spain, consumers’ preferences for olive oil depend more on food traditions than on healthy food choices [23]. In addition, personal traits influence consumers’ utilities; for example, it seems that dietary traditions and food-related activities (such as cooking) are more important than healthy food beliefs for Spanish consumers, and particularly for males [24].

### 2.2. Health Claims Related to Olive Oil Consumption

The existing literature addressed the cognitive processes correlated with the effects of food labels and the role of nutritional labels in driving consumers’ healthy food choices [24,25]. Although information about the nutritional content of food has a strong influence on consumers’ choices of healthier food products [26], in many cases, the nutrition information provided on such labels is underutilized by consumers due to their limited amount of objective knowledge [4,19,25].

In addition, a recent paper pointed to the coexistence of different market segments with different levels of sensitivity to nutrition and health claims, thus identifying potential claim users, actual claim users, and non-users [4]. Conversely, the role of health claims in consumers’ olive oil choices is quite under-researched. Existing studies have argued that health claims could be a useful tool for categorizing different segments of olive oil consumers [20].

A previous study by Finardi et al., (2009) [27] found that consumers had a low willingness to pay for olive oils with health claims. Consumers may not consider health claims to be an attractive driver because they are already conscious of olive oil’s important role in preventing several diseases. This result was also partially confirmed in another study that found that in many cases, the symbols reported on packaging and those related with health claims are more important and appealing than verbal nutritional cues reported on labels [28].

However, not all authors agree regarding the relevance of the role played by labels in consumers’ olive oil choices. Similarly, other authors have explored how consumers’ socio-demographic characteristics may relate to healthy food choices. In a study by Contini et al., (2015) [29] a positive correlation was identified between males and health claims, and young consumers showed a higher propensity towards health claims.

### 2.3. Psychological Factors Related to Consumers’ Preferences: The Influence of Consumer Knowledge

The role of psychological factors on consumers’ behaviour has been widely analysed by many authors, and particular emphasis has been placed on subjective knowledge and self-consistency [11,12].

The role of subjective knowledge in consumers’ choices has been widely debated [11,13,30]. Starting from product knowledge as the preliminary concept in the information-collecting and decision-making process, Brucks (1985) [30] introduced the concept of subjective knowledge in her seminal paper. Subjective knowledge, also known as perceived knowledge, varies as external motivations are consistent with the subjective information of consumers [11]. In addition, subjective knowledge expresses consumers’ familiarity with a product [31].

The influence of subjective knowledge on consumers’ behaviour has been also investigated by analysing food consumers’ attitudes towards different products. Researchers have discussed and analysed the role of subjective knowledge in organic food consumption [13] identifying a positive correlation between the two [13]. The authors determined individual’s attitudes towards organic food by combining their beliefs and values, thus showing that perceived knowledge is associated with self-confidence [13].

The association between nutritional information and health consciousness has also been analysed in other studies on consumer behaviour. One such study, which was based on the acceptance of genetically modified foods, showed that subjective knowledge cannot be considered as a ‘unidimensional construct’; instead, it must fit within the study and the specific product [32]. Another study, however, highlighted the low importance of nutritional knowledge in wine consumers’ choices [33].

Santosa et al. [7] found that the health benefits of olive oil are little known by US consumers, but olive oil is considered to be a genuine and natural product obtained through a physical production process without the use of solvent chemicals [7]. In addition, it has been observed that subjective knowledge has a high explanatory capacity in health-related olive oil consumers’ attitudes and in their consumption frequency [10].

## 3. Materials and Method

### 3.1. The Conceptual Framework

**Figure 1:**
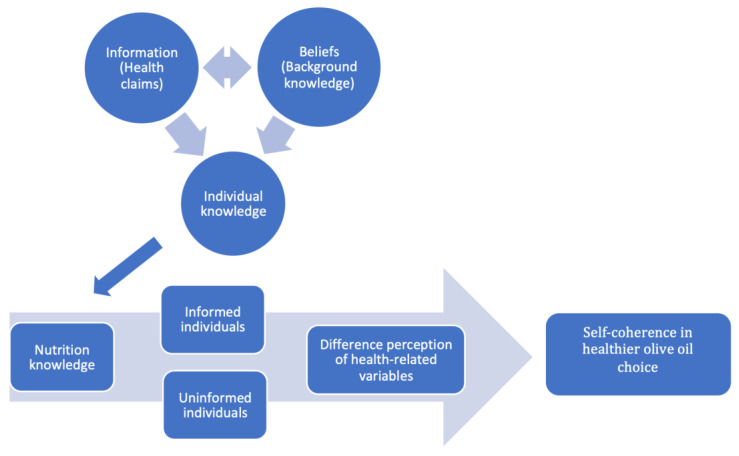
The conceptual model for healthier olive oil choices in informed and uniformed individuals.

In this study, we propose a conceptual framework that combines and integrates two different approaches: the model for healthier food choices introduced by Barreiro Hurle et al. [26] and the theory of subjective knowledge and self-consistency of consumers [11,30,34]. In particular, our conceptual model associates the subjective knowledge of consumers with their attitudes and motivations towards healthy olive oils. Within this context, it is important to understand which characteristics of the product influence the consumption of healthy olive oil (Figure 1).

The conceptual model for healthier food choices developed by Barreiro Hurle et al. [26] consists of three steps: nutritional knowledge leads to nutrition label detection, which finally allows consumers to make healthier food choices. In parallel, subjective or perceived knowledge varies as environmental stimuli are consistent with consumers’ subjective information and educational level and finally with their self-consistency [11].

Prior research has shown the effects of individual knowledge on ‘nutrition search selectivity and choice’, given that the self-consistency helps, directs, and orientates consumers [11,13].

The importance ascribed to the healthiness of olive oil differs among informed or uninformed consumers. This implies that the value associated with healthiness can be associated with few or many different characteristics or attributes.

For this reason, variables were borrowed (derived) from the current literature on healthy food choices, olive oil consumption, and subjective knowledge. In our approach, it is not important whether the consumer has complete or correct information about the health content of olive oil.

Therefore, we aimed to determine whether individual knowledge and the consequential self-coherence of more or less informed individuals leads to different reflections and perceptions of the same product, giving rise to preferences otherwise influenced by different exogenous variables.

Consumers’ knowledge is based on external information and on their own beliefs; they both contribute to forming the individual knowledge that differently guides the attitudes and choices of nutritionally informed or misinformed consumers. Consequently, the differences in consumers’ nutritional knowledge give them different perceptions of the health-related variables of olive oil. Therefore, consumers’ choices of healthier olive oil are strongly dependent on their own self-coherence.

### 3.2. Data Collection

A direct survey was implemented by interviewing a casual sample of 767 regular olive oil consumers. After the selection and validation of the questionnaires, we gathered a total of 708 valid observations, the general characteristics of which are shown in Table 1.

The interviews took place in three different regions of Italy lasting around ten minutes. The respondents were randomly recruited while shopping in large-scale retail hypermarkets [35,36] in the cities of Turin, Milan, and Reggio Calabria. In-store interviews were directly carried out using a specific questionnaire. 

The questionnaire containing open-ended questions was implemented with the face-to-face method. Questionnaires consisted of four main sections. The first section was used to gather information about the respondents’ attitudes and their purchase frequency, motivations, and consumption processes (habits, place of purchase and general attitudes).

The second section included questions about their health perception, nutritional content, and health-related aspects of olive oil consumption. It also aimed to verify the beliefs of consumers on their nutritional knowledge, as reported below in the specific section on experimental design. The third section aimed to explore the importance attributed to the intrinsic and extrinsic attributes of olive oil by taking into account the consumers’ preferences for the sensory profile of olive oil (colour, transparency and taste) and credence attributes such as organic production method, label information, brand. The last part of the questionnaire collected information about the socio-demographic characteristics of the respondents such as gender, age, education, geographical provenance and body mass index (BMI). The BMI index is used to estimate the amount of body fat a person has; it takes into account the ratio between weight, expressed in kilograms and height, expressed in metres (kg/m^2^). According to the World Health Organization, the BMI main classes are identified as follows: <18.5 kg/m^2^: underweight; 18.5–24.9 kg/m^2^: normal weight; 25.0–29.9 kg/m^2^: overweight; 30.0–34.9 kg/m^2^: I obesity.

The questions were organized as binary questions (yes/no answers) in the case of gender and provenance, or using a seven-point Likert scale in order to verify the respondents’ total agreement (7) or total disagreement (1) for all the other remaining variables. For example, concerning the health-related aspects of olive oil, consumers were asked to express their agreement scale, such as: “I believe the consumption of olive oil for is useful for the prevention of the following pathologies: cardiovascular disease prevention” (1 = very low, 7 very high), cancer disease prevention (1 = very low, 7 very high).

### 3.3. Experimental Design

This study examined the stated preferences of consumers according to whether they stated that they had good knowledge about the nutritional characteristics of olive oil. Respondents were subjected to one preliminary question focused on their familiarity with olive oil in which they were asked to state their individual knowledge of olive oil as a product and its nutritional characteristics.

Respondents’ level of consumer knowledge and information was not subjected to pre-screening questions, as has been the process in studies on subjective knowledge theory [11,34]; however, the segmentation of the sample was carried out by giving priority to self-coherence by relying on ex post evaluations using inferential statistics.

Starting from previous research on subjective knowledge about agri-food products [10,13,31] and considering consumers’ opinions, we assessed the level of their knowledge about olive oil through their responses to the following binary question (yes/no) contained in the questionnaire:

‘I believe that I know the nutritional properties of olive oil’.

The critical choice in dichotomising self-declared knowledge was justified insofar as the consumers’ stated individual knowledge influenced their perceptions of the healthiness of olive oil. Consequently, the same information, indicating high polyphenol content, conveys different healthiness cues to informed and uninformed consumers.

Consumers who considered themselves to be informed answered “yes” to this question. Conversely, uninformed consumers replied no to the preliminary question.

Subsequently, two sub-samples were generated on the basis of the respondents’ self-declared knowledge about the nutritional properties of olive oil. The first group, highly informed consumers, included 267 respondents that declared that they had a medium-high level of knowledge.

The second group, less informed consumers, included 441 respondents with a limited self-declared degree of knowledge about olive oil and its nutritional properties.

Our investigation attempted to analyse the respondents’ perceptions and identify differences between these two groups. Therefore, the respondents evaluated the health properties of olive oil by assigning it with a rating, since food can evoke different emotional and cognitive expectations in different individuals.

### 3.4. Inferential Statistics

Obtained results were analyses applying bivariate inferential statistics to verify the effective differentiation and to validate the existence of two significantly different groups. To this end, independent sample t-tests and Pearson chi-square tests for socio-demographic characteristics were employed [37]. In addition, age was tested by t-test because it is a continuous variable. The t-test allows the verification of a significant difference between the average values of two groups with respect to the investigated variables [38], while the chi-square test allows the determination of whether two categorical variables are independent or associated [39].

The attributes were chosen by reviewing the most relevant literature on olive oil consumer behaviour and selecting variables that could have directly or indirectly affected respondents’ choices of an olive oil with a high polyphenol content as shown in Table 2.

Once it was verified that significant differences existed between the two groups of informed and uninformed consumers, these variables were included as covariates in the econometric model, as described in the following paragraph.

### 3.5. The Econometric Model

The econometric model reflects the relationship between the endogenous variable, namely the willingness to buy olive oil with high polyphenol content, and the groups of independent variables that may have had an effect on healthier olive oil choices. Two econometric analyses were carried out to identify the drivers of the consumption of olive oil with a high polyphenol content for each group differentiated on the basis of their individual knowledge.

Two logit models were generated and an analysis of marginal effects was subsequently carried out.

We chose a logit model as the tool to answer the research questions [19]. In the model, the dependent variable is dichotomous—value 1 corresponds to the respondents’ positive willingness to buy olive oil with high polyphenolic content, while value 0 represents a negative propensity.

The covariates were selected by analysing the existing literature on healthy olive oil consumption. The tested covariates included the intrinsic and extrinsic attributes of olive oil as well as the socio-demographic characteristics of the sample. In addition, some interaction variables such as Age*Cardiovascular disease, Age*Bitter, Gender* Cardiovascular disease and Gender*Bitter were included.

To ensure that generated model was not too large, thus not losing degrees of freedom, the numbers of interaction terms were reduced to be consistent with the aims of the survey [47]. For these reasons, we combined two highly relevant socio-demographic characteristics, age and gender, with two fundamental variables related to the healthy consumption of olive oil, namely, ‘bitter taste’ and the ‘prevention of cardiovascular disease’ [10,43].

Subsequently, in order to select the variables that significantly contribute to the consumer’s choice and to obtain a robust model through an iterative selection process [48], a stepwise selection using the backward method was carried out, similarly to methods used in the literature [49].

Once the final models were obtained, the presence of multicollinearity between the covariates selected using the stepwise process was evaluated by means of a variance inflation factor (VIF). After checking the absence of multicollinearity, we tested the goodness-of-fit of the final models by the means of McFadden’s Pseudo r-square and the Log likelihood.

Finally, we analysed the marginal effects. This was necessary to complete the information provided by the logistical analysis. In fact, the coefficients obtained from the analysis were not directly interpretable for the quantification of the effects. To overcome this problem, the study of marginal effects can be a useful tool for evaluating how the variation of the covariates can influence the probability of the dependent variable to change from 0 to 1 [19]. The analysis was carried out using Stata 15.1.

## 4. Results and Discussion

### 4.1. Descriptive Analysis

This section reports the main results of the descriptive analysis. Table 3 shows the socio-demographic characteristics of two sub-samples—informed consumers (*n* = 267) and uninformed consumers (*n* = 441)—and reports the results of the inferential test employed to verify the existence of statistically significant differences between informed and uninformed consumers.

The average age of the first group was higher than that of the second one; in fact, the average age of the informed consumers was 45.28 years old, while that of the uninformed was 43.00, and the difference between them was significant according to the *t*-test.

With respect to gender, Table 4 shows an increased prevalence of males compared to females in both sub-samples; however, the chi-square test does not show significant differences between the two groups.

There was a larger presence of highly educated individuals among the group of informed consumers. This group was mainly represented by consumers from northern Italy, while the group of uninformed consumers was mainly from southern Italy.

Finally, concerning the average BMI, the index for both consumer groups was distributed in a similar manner between different classes and no significant differences existed. The results of inferential tests thus show that the sub-groups were significantly differentiated in terms of socio-demographic characteristics.

Similarities and differences were identified considering socio-demographic features with respect to the existing literature. In fact, although nutritional knowledge [5,26] and the perception of the health of olive oil [14,22] have been found to be positively influenced by education, gender, income, and health status, although studies on subjective knowledge have pointed out the inconsistent relationship with socio-demographic characteristics such as age, education, and income [13], we only found the age and the regional origin of consumers to be significant. Concerning age, the findings of the current study are consistent with previous finding [22], especially considering elderly consumers’ low perception of the ability of olive oil to reduce disease.

Table 4 reports the descriptive analysis of consumers related to the intrinsic and extrinsic attributes and the results of the t-test. It is notable that almost all of the variables show statistical differences between the groups in the t-test. Consequently, this preliminary test highlighted a significant diversity between the two sub-samples, with the only exception for the transparency, and supports the intuition that two econometric analyses could be developed in order to understand how these variables influence the behaviour of these consumers.

Considerations on inferential tests can be provided. For example, it’s notable that bitter taste and pungent attributes that are directly related to high polyphenol content [20] are considered more important among informed consumers. On the contrary, label information is an attribute that is more requested on average by uninformed consumers. Finally, the health aspects of olive oil and its taste have quite similar importance for both groups of respondents.

### 4.2. Logistic Regression Analysis

In this section, we report the main outcomes of the two logistic models, as shown in Table 5. The results obtained for the two groups of consumers are quite different as evidenced by the different covariates that significantly influence the consumers’ preferences.

Informed consumers believe that bitter taste is positively and significantly linked to the consumption of olive oil with high polyphenol content; this is an accurate association [15]. Therefore, as the importance ascribed to bitter taste increases, informed consumers are more likely to buy olive oil with high polyphenol content. This result is consistent with authors who observed a high propensity to consume bitter olive oil among knowledgeable consumers [42].

Equally significant for informed and uninformed consumers is the variable ‘positive action on immune system’, since this variable positively contributes to the purchase of olive oil with a high polyphenol content. This result indicates that the healthiness of olive oil is considered to be a driver for olive oil consumption for both groups, thus confirming in previous study regarding health benefit perceptions among olive oil consumers [7].

In addition, it can be seen that geographical origin plays a significant role only for informed consumers. Specifically, consumers in southern Italy have a greater propensity to perceive olive oil as a healthy product. This appears to be in line with previous analysis on southern Italian consumers [45].

Considering the interaction variables, it can be inferred that the relations Age*Cardiovascular disease is significant for both informed and uninformed consumers, while Age*Bitter is significant only for informed consumers. The first interaction, Age*Cardiovascular disease, suggests that the simultaneous increase in age and in the perception of the ‘heart disease prevention’ may increase consumers’ propensity to buy such products. This is reasonably explicable because older people are more interested in ‘healthy eating’ than younger people [25]. Regarding the values of the coefficients, this variable is similarly important for informed and uninformed consumers.

The second interaction variable, Age*Bitter, is negatively related with the consumption of these products only for informed consumers. The negative coefficient associated with these attributes suggests that a concomitant increase in age and in bitterness implies a decreasing attitude towards olive oils with high polyphenol content. This is rather consistent with studies regarding sensitivity towards bitterness, as younger consumers were found to be more sensitive, since a decrease in bitterness sensitivity with age has been found [50,51].

Regarding the importance of the transparency of olive oil, the model indicates that uninformed consumers ascribe importance to the clarity of olive oil. This may imply that such consumers are not aware that a clearer olive oil may contain fewer substances in suspension, including polyphenols. In fact, filtration can increase the stability and shelf life of olive oil but sacrifices a certain number of phenolic compounds [52].

Thus, although transparency did not show significant differences between the averages of the two groups, the t-test (Table 4) indicated that this attribute is significant in the logistics model for uninformed consumers. This apparent discrepancy is due to the fact that the t-test is used exclusively to highlight differences between the mean values of different groups [38], while the logistics model predicts that a certain event will happen [53].

For uninformed consumers, another important role is played by overall taste. This covariate is negatively associated with the consumption of olive oil with a high polyphenol content, probably because uninformed consumers do not identify a direct relationship between the taste and polyphenol content. This result is in line with a previous study in which the global indicator of the sensory profile of olive oil was negatively correlated with enjoyment by regular consumers [43].

The role of label information in the literature is quite controversial; many authors argue that its role is important in olive oil consumers’ choices [29], while other authors find that label information is negatively related to consumers’ choices [43]. Although there is no shared and univocal view on the influence of label information on the consumption of olive oil, our findings reveal that label information is only important for uninformed consumers, since it is positively correlated with a healthy product. This could indicate that less informed consumers use labels as a quality signal to detect information about the health components of olive oil.

Age is the variable of least interest but only in the case of uninformed consumers. Its negative coefficient indicates that as the age of respondents increases, interest in healthy olive oil decreases. This result partially confirms the results of a study conducted on nutraceutical olive oil, which argued that older people attach less importance to health claims in favour of aspects related to traditions and the territory [22].

Table 6 shows the results of the variance inflation factor (VIF) analysis and highlights the absence of multicollinearity among the selected variables. This indication is provided by the 1/VIF ratio. In fact, when the ratio is greater than 0.2, multicollinearity can be considered irrelevant because the stability of the coefficients provided by the logistic model is satisfactory [54].

### 4.3. The Analysis of Marginal Effects

As stated previously, the coefficients of the logistic model cannot be directly interpreted to quantify the effects of the covariates. In fact, through the coefficients of the model, it is only possible to recognize the direction of the effect indicated by the sign. The analysis of marginal effects shown in Table 7, on the other hand, allows us to assess how the marginal variation of an independent variable could modify the outcome holding the other mean value of regressors constant. As the dependent variable is a dummy, the study of the marginal effects makes it possible to assess the probability of the dependent variable in a range between 0 and 1 [19].

The main explanatory variables of the informed consumers’ stated preferences are bitter taste, health benefits, and the consumer’s geographic origin.

The unitary variation of bitter taste causes an increase in the probability of purchase at 11%, while the unitary variation of the variable ‘positive action on immune system’ produces an increase in the probability of purchase at 4.7%. Regarding consumers’ geographic origin, which is expressed as a dichotomous variable, changing the variable from 0 to 1, where 1 corresponds to southern Italian consumers, causes an increase in probability of 17.6%.

For uninformed consumers, transparency is one of the variables positively associated with the consumption of healthy olive oils. The probability that such consumers will buy healthy olive oils increases by 2% for each unitary variation on the Likert scale. Regarding the other variables positively associated with healthy olive oil consumption, a unitary variation on the Likert of the covariates ‘Label information’ and ‘Positive action on immune system’ causes an increase in the probability of buying healthy olive oil by 3.4% and 4.2%, respectively.

Focusing on the variables negatively correlated with consumption, the unitary variation of overall taste causes a decrease in the probability of consuming healthy olive oils by 4%. Finally, concerning age, the marginal effects of ageing are represented by a decrease in the probability of consuming functional olive oil by 0.5% for each year.

Scholars have identified consumers’ high willingness to buy olive oils with functional claims [22], but healthy olive oil production still covers a niche market.

This study contributes to enriching the current state of expertise on consumers’ knowledge about healthy foods. The aim of this study was to evaluate the consumers’ attitudes towards extra-virgin olive oil with high polyphenol content by assessing whether the perception of olive oil as healthy differed among informed or uninformed consumers.

This study verified that, even for healthy foods, consumers’ choices are strongly dependent on their own self-coherence. As a consequence, consumers’ knowledge or beliefs that orientate their attitudes are influenced by different motivations and attributes.

The outcomes of the present study support the initial hypothesis that individual knowledge has different effects on consumers’ preferences for healthy olive oil. As a consequence, in response to the research question, the variables that influence low- and high-knowledge consumers’ attitudes towards healthy olive oil are substantially different, although it is worth noting that similarities exist, such as perceptions regarding the healthy and beneficial properties of olive oil. Thus, the same label may convey different health cues according to the level of consumers’ self-declared knowledge. Consumers modify their perception of bottled olive oil with a high polyphenol content according to their degree of information.

Finally, it is worth noting that the variables that were found to be significant in the model are consistent with the level of consumer knowledge and subsequently, differences in self-coherence between the two groups of consumers (less or more informed) seems to be confirmed.

We can reasonably affirm that different beliefs and knowledge can lead to the choice of the same product with different additional characteristics. That is, even if consumers choose the same health product, the variables that influence informed and uninformed consumers are substantially distinct.

We found that several motivations for consumers’ behaviour are consistent with their stated subjective knowledge given that self-consistency differently addresses nutrition search selectivity and choice in the buying process [12]. This confirms that in many cases, consumers’ subjective knowledge may result in the translation of their attitudes into preferences [13].

Regarding the specific group of variables associated with the beneficial effects of olive oil, our study found empirical evidence for the three groups of attributes as follows: sensory variables such as transparency, bitter taste, and taste as a whole; credence and health property variables (label information regarding benefits for the immune system); and socio-demographic characteristics (consumers’ age and geographic origin).

Consistent with previous studies on the importance of bitter taste as an indicator of the healthiness of olive oil [55], our study confirms the importance that the informed consumer gives to this attribute, which is not typically considered a preferred attribute. Conversely, uninformed consumers incorrectly believe that transparency is an attribute related to health benefits, although it is commonly known that turbid olive oils contain more substances in suspension, including polyphenols [51]. At the same time, as was expected, only consumers with a low knowledge level associated an olive oil’s taste in its entirety with healthiness.

Concerning the credence and health properties, consumers’ different degrees of information did not always lead to different perceptions. Different effects of individual knowledge were found regarding label information. Only uninformed consumers believed that label information was important, which is a reasonable consequence of their lack of information.

Conversely, taking this latter credence attribute into account, outcomes regarding the health benefits of olive oil are rather similar between two groups. Indeed, the groups’ perceptions did not differ regarding the variables ‘positive action on immune system’ and the interaction variable ‘Age*Cardiovascular disease’. This confirms the widespread beliefs among consumers regarding the beneficial properties of olive oil consumption.

At the same time, for the first time, a direct association was found between the consumers’ knowledge and their geographical origins. However, this result cannot be generalized because it concerns consumers who live in traditional olive oil-producing areas.

Finally, the nutritional and sensory attributes, with the exception of bitter taste, are viewed as more important by low-knowledge consumers. Conversely, high-knowledge consumers attach more importance to health attributes, being also characterized by ageing, thus confirming that intrinsic attributes rise in importance as the level of knowledge, and as such the information, rise [27].

## 5. Conclusions

This study highlighted that the individual knowledge about functional olive oil is strongly influenced by different attributes, and as a consequence, the same label may convey different health cues for informed and uninformed consumers.

This study provides useful insights for olive oil market practitioners, as the health benefits of olive oil are progressively gaining a global consensus among academicians and purchasers. Consequently, consumers’ perceptions and motivations are crucial both for public policy and business strategies. This suggests the importance of disseminating health information and conducting promotion campaigns through public and collective action. In addition, consumers’ knowledge about the health effects of the polyphenols in olive oil is rather restricted, and our study highlights the need to enhance the information about the benefits of polyphenols.

The findings of this study also have implications from a marketing perspective, since the different variables that influence consumers’ choices of healthy products allow for the identification of different market segments. Consequently, several market segments can be identified according to the consumers’ level of information; for this reason, producers should differentiate their information campaigns regarding the health values of functional olive oil.

This study has some limitations due to the limited geographical area of investigation, which is restricted to a single national context, and because of the typology of the recruited convenience sample. Further studies are required to better address these insights. For instance, the experiment could be replicated for other food products or new evidence could be provided by assessing other attributes of olive oil.

## Figures and Tables

**Table 1:** Socio-demographic statistics of the validated sample (*n* = 708).

Variable	Value
Age: average-(SD)	43.86 (13.81)
Gender	
Female	44.6%
Male	55.4%
Level of education	
Elementary school	2.8%
Medium school	22.3%
High school	41.9%
Degree	29.7%
Post degree	3.2%
Provenance	
North Italy	44.6%
South Italy	53.4%
Body Mass Index	
Under weight	2.4%
Normal weight	57.9%
Over weight	30.9%
Obesity class I	8.8%

**Table 2:** Tested attributes and socio-economic characteristics of the sample.

Variable	Type	Reference
Green colour	Cat (1-7)	[40]
Transparency (clear)	Cat (1-7)	[41]
Bitter taste	Cat (1-7)	[42]
Pungent taste	Cat (1-7)	[42]
Overall taste	Cat (1-7)	[43]
Organic label	Cat (1-7)	[40]
Label information (nutritional)	Cat (1-7)	[43]
Anti-inflammatory activities	Cat (1-7)	[44]
Positive action on immune system	Cat (1-7)	[7]
Prevention of cardiovascular disease	Cat (1-7)	[10]
Age	Continuous	[22]
Gender	Dummy	[42]
Education	Cat	[42]
Provenance (north/south)	Dummy	[45]
Body Mass Index (BMI)	Cat	[46]

**Table 3:** Bivariate descriptive statistics for socio-demographic variables.

	Aware Consumers (267)	Non-Aware consumers (441)	Test
Variable	Value	Value	*p*-value
Age: average-(SD)	45.28 (14.00)	43.00 (13.63)	0.033 **
Gender			0.168
Female	47.9%	42.6%	
Male	52.1%	57.4%	
Level of education			
Elementary school	2.6%	2.9%	0.000 ***
Medium school	13.5%	27.7%	
High school	39.7%	43.3%	
Degree	37.8%	24.7%	
Post degree	6.4%	1.4%	
Provenance			
North Italy South Italy	77.5% 22.5%	27.9% 72.1%	0.000 ***
BMI			
Under weight	2.2%	2.5%	0.758
Normal weight	59.9%	56.7%	
Over weight	30.3%	31.3%	
Obesity class I	7.5%	9.5%	

*, ** and *** denote significance at 10%, 5%, and 1% levels, respectively.

**Table 4:** Bivariate descriptive statistics for the intrinsic and extrinsic attributes of olive oil.

	Aware Consumers (267)	Non-Aware Consumers (441)	Test
Variable	Mean (SD)	Mean (SD)	*p*-value
Green colour	4.73 (1.71)	4.37 (1.96)	0.013 **
Transparency (Clear)	3.96 (1.89)	3.92 (2.06)	0.812
Bitter taste	3.79 (2.04)	3.26 (2.03)	0.001 ***
Pungent taste	4.41 (1.86)	3.56 (1.99)	0.000 ***
Taste	5.81 (1.51)	6.00 (1.25)	0.076 *
Label information	5.36 (1.68)	5.76 (1.44)	0.001 ***
Anti-inflammatory activities	4.45 (1.82)	4.04 (2.03)	0.008 ***
Positive action on immune system	4.14 (1.16)	4.32 (0.96)	0.032 **
Prevention of cardiovascular disease	5.25 (1.71)	5.49 (1.67)	0.073 *

*, **, and *** denote significance at 10%, 5%, and 1% levels, respectively.

**Table 5:** Logistic regression of the attributes affecting the consumption of olive oil with a high antioxidant content.

	Informed Consumers (267)		Misinformed Consumers (441)	
Variable	Coefficient	*p*-Value	Coefficient	*p*-value
Transparency			0.137	0.018 **
Bitter taste	0.537	0.002 ***		
Taste			−0.231	0.025 **
Label information			0.195	0.014 **
Positive action on immune system	0.226	0.056 *	0.240	0.048 **
Age			−0.044	0.001 ***
Provenance	0.974	0.034 **		
Age* Cardiovascular disease	0.004	0.021 **	0.005	0.004 ***
Age*Bitter	−0.009	0.005 ***		
Constant	−1.671	0.008 ***	0.661	0.454
Log likelihood	−150.292		−227.614	
Pseudo R2	0.100		0.069	

*, **, and *** denote significance at 10%, 5%, and 1% levels, respectively.

**Table 6:** Variance inflation factor (VIF) analysis performed for both logit models: informed and uninformed.

	Informed Consumers (267)		Uninformed Consumers (441)	
Item	VIF	1/VIF	VIF	1/VIF
Transparency (clear)	-	-	1.02	0.983
Bitter taste	1.10	0.909		
Taste	-	-	1.09	0.917
Label information	-	-	1.06	0.941
Positive action on immune system	1.07	0.933	1.12	0.895
Age			1.01	0.986
Provenance	1.14	0.874		
Mean VIF	1.11		1.06	

**Table 7:** Marginal effects of attributes affecting the consumption of olive oil with high antioxidant content.

	Aware Consumers (267)		Non-Aware consumers (441)	
Variable	Coefficient	*p*-Value	Coefficient	*p*-Value
Transparency			0.024	0.017 **
Bitter taste	0.110	0.002 ***		
Taste			−0.040	0.024 **
Label information			0.034	0.014 **
Positive action on immune system	0.047	0.056 *	0.042	0.047 **
Age			−0.008	0.001 ***
Provenance	0.176	0.011 **		
Age * Cardiovascular disease	0.001	0.021 **	0.001	0.003 ***
Age*Bitter	−0.002	0.005 ***		

*, **, and *** denote significance at 10%, 5%, and 1% levels, respectively.
